# Microbial Diversity of Genital Ulcer Disease in Men Enrolled in a Randomized Trial of Male Circumcision in Kisumu, Kenya

**DOI:** 10.1371/journal.pone.0038991

**Published:** 2012-07-27

**Authors:** Supriya D. Mehta, Stefan J. Green, Ian Maclean, Hong Hu, Robert C. Bailey, Patrick M. Gillevet, Greg T. Spear

**Affiliations:** 1 Department of Epidemiology and Biostatistics, University of Illinois at Chicago School of Public Health, Chicago, Illinois, United States of America; 2 DNA Services, Research Resources Center, University of Illinois at Chicago School of Medicine, Chicago, Illinois, United States of America; 3 Department of Medical Microbiology, University of Manitoba, Manitoba, Winnipeg, Canada; 4 Department of Bioinformatics, University of Illinois at Chicago, Chicago, Illinois, United States of America; 5 Department of Environmental Sciences and Policy, George Mason University, Fairfax, Virginia, United States of America; 6 Department of Immunology and Microbiology, Rush University Medical Center, Chicago, Illinois, United States of America; Karolinska Institutet, Sweden

## Abstract

**Background:**

Medical male circumcision (MMC) reduces the risk of genital ulcer disease (GUD) in men by 50%. In Ugandan and Kenyan trials, a sexually transmissible agent was not identified in 50–60% of GUD specimens by polymerase chain reaction (PCR) assay. We sought to better define the etiology of GUD in men participating in the Kenyan trial and examine how MMC affects GUD etiology.

**Methods:**

We defined GUD of unknown etiology as negative for HSV (type 1 and type 2), *T. pallidum*, and *H. ducreyi* by PCR, and negative for HSV-2 and *T. pallidum* by serology. We identified bacterial microbiota in a subset of 59 GUD specimens using multitag pyrosequencing of the 16S rRNA gene, and compared results by unknown vs. STI-associated etiology. Statistical analysis employed Bray-Curtis similarity measure of bacterial community by etiology, hierarchical clustering and logistic regression.

**Results:**

In 59 GUD specimens from 59 men, 23 (39%) had unknown etiology. Bacterial diversity was greater in GUD of unknown than STI etiology (p = 0.01). Fusobacteria (*Fusobacterium* spp. and *Sneathia* spp.) were more commonly detected in men with GUD of unknown etiology [adjusted OR = 5.67; 95% CI: 1.63–19.8] as were *Oxobacter* spp. and *Anaerovorax* spp. [adjusted OR = 3.12; 95% CI: 0.83–11.7]. Sequences from these four anaerobic bacterial taxa were more often detected in uncircumcised men than circumcised men (p<0.05).

**Conclusions:**

Anaerobic bacteria are more common in genital ulcers of uncircumcised men. The specific anaerobic bacteria associated with GUD of unknown etiology have cytotoxic properties that can exacerbate epithelial disruptions leading to ulcer-like appearance. MMC may reduce GUD through a reduction in these anaerobic bacteria.

## Introduction

In three randomized trials, adult male medical circumcision (MMC) reduced the risk of HIV acquisition in African men by approximately 60% [1]–[3]. In the MMC trial in Uganda, circumcision resulted in a 46% reduction in GUD [2] and a 28% reduction in *Herpes simplex* virus type 2 (HSV-2) acquisition [4], similar to the 30% reduction in HSV-2 incidence observed among MMC trial participants in South Africa [5]. In the trials in South Africa and Uganda protection against HIV was in part mediated through a reduction in HSV-2 [5]–[6]. In contrast, in the Kenyan MMC trial, MMC led to a 50% reduction in GUD, but did not reduce HSV-2 incidence [7]. In both the Ugandan and Kenyan trials, multiplex polymerase chain reaction (PCR) failed to identify a sexually transmissible etiologic agent in 50–60% of genital ulcer swab specimens [6]–[7]. Without knowing the cause of these genital ulcers, it is difficult to understand the mechanism by which circumcision confers protection against them.

Preventing and treating GUD is relevant to maximizing HIV prevention. HSV-2 and GUD increase HIV acquisition by 2–6 times among men and women 8–10. Independent of circumcision status and HSV-2 infection, the risk of HIV seroconversion among men in our cohort in Kenya was 7 times greater for men with GUD [7]. Despite the consistent association between HSV-2 and HIV risk in observational studies, a randomized trial of daily acyclovir treatment for HSV-2 and HIV- co-infected individuals did not find a protective affect against HIV transmission to partners [11], perhaps due to the lack of reduction in non-herpetic ulcers: in 44% of genital ulcers in this study, HSV-2 was not detected. Among HIV-positive men enrolled in a randomized trial of episodic acyclovir, the odds of HIV viral shedding from HSV-2 positive ulcers were 40% lower compared to ulcers with unknown etiology [12].

Lack of identification of HSV-2 or other STIs in GUD may be attributed to sensitivity of assays, specimen collection, or stage of disease, but alternative explanations have rarely been considered. Because of the continuing contribution of GUD to the HIV epidemic in sub-Saharan Africa and the failure of HSV-2 suppressive therapy to prevent HIV transmission, it is essential to determine if a broader range of infectious pathogens is involved in inducing GUD so that effective GUD treatment and prevention measures can be developed. We conducted an analysis of genital ulcer specimens to identify bacteria potentially associated with GUD etiology through multitag pyrosequencing of the bacterial small subunit (SSU) ribosomal RNA (rRNA) genes.

## Methods

The study was approved by the Institutional Review Boards of the University of Illinois at Chicago, the Kenyatta National Hospital, RTI International, University of Manitoba, Rush University Medical Center, and was overseen by a Data and Safety Monitoring Board. Our trial in Kisumu, Kenya enrolled 2,784 men aged 18–24 years. Trial recruitment, enrollment, reasons for refusing enrollment, and follow-up have been previously described [3] (registration at http://www.clinical trials.gov, number NCT0005937). For inclusion men had to be: uncircumcised, HIV-negative, sexually active in the last 12 months, and aged 18–24 years; have a hemoglobin >9.0 mmol/L; and reside in Kisumu District. Exclusion criteria included: foreskin covering less than half of the glans, a bleeding disorder, keloid formation, other conditions that might unduly increase the risks of elective surgery, or a medical indication for circumcision. Following written informed consent, participants were randomized 1∶1 to either immediate circumcision or delayed circumcision (the control group) after a two-year follow-up period. Testing for HIV was conducted at baseline, 1, 3, 6, 12, 18 and 24 months from randomization for both the circumcision and the control groups, and physical examination and behavioral surveys occurred at baseline and every 6 months thereafter.

### Physical Examination Findings of Genital Ulcer Disease

All consenting participants underwent standardized medical examination and history [3]. At each planned study visit, genital examination by trained clinicians recorded the presence or absence of genital ulcers, and the location and number of ulcers. The same exam form was used to record findings from men who presented between study visits with genital complaints.

### Detection of HSV-2, Treponema pallidum, and Haemophilus ducreyi in Genital Ulcers

Serum specimens were tested for HSV-2 antibody (Kalon HSV-2 IgG ELISA, Kalon Biological Limited, Aldershot, United Kingdom), using the manufacturer's recommended cut-off. Syphilis infection was detected using the rapid plasma reagin test (RPR) (Macro-Vue™, Becton Dickinson, New Jersey, United States), confirmed by the *T. pallidum* hemagglutination (TPHA) assay (Randox Laboratories Ltd., Ardmore, United Kingdom). All genital ulcers were tested for *H. ducreyi* by culture. Testing was conducted at the study clinic. A convenience sample of 94 (35%) specimens of 266 clinically identified genital ulcers detected between March 2002 through February 2007 were tested for *H. ducreyi*, *T. pallidum* and HSV (did not distinguish between type 1 and type 2) by multiplex polymerase chain reaction (PCR) at the University of Manitoba Department of Medical Microbiology research laboratory. Characteristics of the 94 men whose GUD specimen was selected did not differ from the characteristics of the 172 who were not selected by baseline treatment assignment, syphilis or HSV-2 serostatus, age, marital status, number of sex partners, condom use, or penile hygiene. However, of the 94 GUD specimens selected for PCR, 37% were from the baseline, pre-randomization visit compared to 23% in the total sample of clinically detected ulcers.

### HIV Testing

Testing for HIV infection was conducted using a parallel double rapid test protocol, using Determine® HIV 1/2 (Abbott Diagnostic Division, Hoofddorp, The Netherlands), and the Uni-Gold Recombigen™ HIV Test (Trinity Biotech, Wicklow, Ireland)^1^. Concordant positive results were confirmed by double ELISA and line immunoassay (INNO-LIA HIV 1/2, Immunogenetics NV, Ghent, Belgium). Specimens indeterminate by line immunoassay were tested by PCR at Health Canada or the Fred Hutchinson Cancer Research Center (Seattle, WA, USA), with the PCR result deemed to be definitive.

### Specimen Selection for Pyrosequencing

Due to resource constraints, a random sample of 65 specimens was drawn from the 94 specimens that underwent multiplex PCR; 59 (91%) had amplifiable bacterial DNA. Characteristics of the 65 men with specimen selection for pyrosequencing did not differ from those 29 that were not selected by circumcision status, socio-demographics, behavioral risks, genital hygiene, HIV status, recovery of HSV or *T. pallidum* from the ulcer, or serological detection of HSV-2 or *T. pallidum*. As expected, the 59 specimens tested differed from the total sample of clinically detected ulcers, whereby 37% were from baseline, pre-randomization visits.

### Multiplex PCR and Multitag Pyrosequencing

At planned or interim visits where genital ulcers were visibly detected by clinicians, a specimen was obtained using Dacron or polyester tipped swabs. If the sores were crusted, the scab was removed to swab the interface between the ulcer and the healthy skin. Specimens were frozen at −20°C, and shipped periodically to the University of Manitoba for detection of HSV, *T. pallidum*, and *H. ducreyi* by multiplex PCR. Swabs were treated with Roche Diagnostics lysis buffer to release the DNA. The specimen was vortexed for 15 seconds in 500 µl of Roche lysis buffer. 100 µl of a Roche diluent (6 mM MgCl2) was added at a 1∶1 ratio by volume to neutralize the SDS. 50 µl of this 200 µl 1∶1 mix was used for the PCR reaction, leaving 150 µl of lysed specimen for pyrosequencing. Multitag pyrosequencing was performed as described previously [13]. Briefly, 12 sets of domain-level primers (*i.e.* 27F or 355R; [13]) containing a four-base barcode were used to amplify the V1 and V2 hypervariable regions of bacterial SSU rRNA genes from genomic DNA extracts. Individual reactions with distinct barcodes were pooled, and Roche 454 pyrosequencing adapters were ligated prior to emulsion PCR and sequencing [13]. Pyrosequencing of the amplified, tagged DNA was performed by 454 Life Sciences (Branford, CT) using titanium technology. From 59 GUD specimens we obtained 100,098 sequences with an average read-length of 346 bases. Sequences shorter than 150 bases were removed from the analysis. An average of 1,697 sequences were obtained from each sample (min = 466, max = 4,015). Bacterial taxa were identified by entering sequences into the Ribosomal database project (RDP; release 10) classifier, using an 80% bootstrap cutoff value [14]. A random sub-sampling of 350 sequences (75% of the smallest dataset) from each sequence library was performed and used for diversity calculations to control for effects of library size [15]. Pyrosequence data were processed through the RDP pyrosequencing pipeline, with a 97% similarity threshold used for clustering of sequences into operational taxonomic units (OTUs) for diversity calculations [14].

### Statistical Analysis

The goal of this analysis was to examine whether specific bacteria were associated with GUD of unknown etiology. GUD of unknown etiology was defined as having no pathogen detected by multiplex PCR in conjunction with no serological evidence of HSV-2 or *T. pallidum*. The analysis proceeded in three steps. First, we compared bacterial communities by STI-related or unknown etiology (step 1). Comparisons of bacterial community (step 1) were quantitatively assessed by Bray-Curtis dissimilarity matrix and Shannon-Wiener diversity index. Relative abundance data were transformed [log(X+1)] prior to generation of the resemblance matrix. Next, to reduce dimension of explanatory variables and identify bacteria that were potentially acting in groups we conducted hierarchical clustering analysis (step 2). Hierarchical clustering was conducted using Spearman rank correlation with complete linkage and groups were identified based on correlations >0.5 (step 2). Individual bacteria identified in step 1 and bacteria grouped by findings in step 2 were examined for association with ulcer etiology. The association between bacteria and ulcer etiology were analyzed as presence, relative abundance, and abundance cutoffs (>1% and >10%) that were comparable to the literature [16]. Finally, along with socio-demographics, circumcision status, and behavioral factors, individual and grouped bacteria that were associated with ulcer etiology at the p≤0.10 level were entered in multivariable logistic regression analysis to identify independent associations between bacteria and unknown ulcer etiology (step 3). We used circumcision status rather than treatment assignment to accurately account for foreskin status as this is demonstrated to be a major mediator of the penile microbiota [16]. All behavioral variables were visit-matched to the visit at which GUD was detected, except age which was measured from baseline prior to randomization.

Differences between continuous variables and outcome were assessed by Wilcoxon rank sum test. Differences between categorical explanatory variables and the outcome were assessed by the chi-square test, or Fisher's exact test when cell size was <5. Variables significant at the p<0.10 level by likelihood ratio testing from univariate logistic regression were entered into multivariable logistic regression. Those variables with a likelihood ratio p-value <0.10 were maintained in the multivariable model. Wald test p-values are presented for the final multivariable model. Comparison of bacterial community composition and hierarchical clustering with generation of dendrograms were conducted in Primer 6.0 (Primer-E, version 6.1.13, United Kingdom). Inferential analyses were conducted using STATA/SE 11.0 for Windows (Stata Corp., College Station, TX).

## Results

In the 59 GUD specimens from 59 men, HSV or *T. pallidum* were detected in 22 (37.3%): 11 with HSV by PCR (and HSV-2 by serology), 7 with HSV by PCR only, 1 with HSV and *T. pallidum* by PCR, and 3 with *T. pallidum* by PCR (1 with syphilis and HSV-2 by serology). In addition to the 22 specimens with HSV or *T. pallidum* detected directly from the ulcer, 14 men were HSV-2 seropositive. Therefore, no etiology was identified by PCR or serology in 23 (39.0%) of 59 men with visibly detected GUD.

### Socio-Demographic and Behavioral Characteristics of Men by Ulcer Etiology

Overall, 9 (15.3%) men were circumcised and 5 (8.5%) were married or cohabiting (Table 1). Men with unknown ulcer etiology were more likely to be aged 21–24 than 18–20 at baseline than those with STI-associated ulcers (65.2% vs. 33.3%, p = 0.02). Circumcision status, marital status, HIV serostatus, number of sexual partners, condom use at last intercourse, other sexual behaviors, and anatomic location of the ulcers did not differ by whether ulcers were of unknown or STI-related etiology. Reported injuries to the penis during or after sex were more common among men with ulcers of unknown etiology than with STI-related etiology, though this approached statistical significance only for reporting the penis ever feels sore after sex (66.7% among unknown etiology vs. 44.1% among STI etiology, p = 0.10).

**Table 1:** Sociodemographic and Behavioral Characteristics by Genital Ulcer Etiology.

Variable		Unknown Etiology, N = 23	STI-Related Etiology+, N = 36	*P-value
		n (%)	n (%)	
Circumcised		3 (13.0)	6 (16.7)	>0.99
Age in years at Baseline	18–20	8 (34.8)	24 (66.7)	
	21–24	15 (65.2)	12 (33.3)	0.02
Married or cohabiting		1 (4.6)	4 (12.1)	0.64
HIV seropositive		3 (13.0)	1 (2.8)	0.16
Number of sex partners, past 30 days	0	5 (22.7)	9 (26.5)	0.78
	1	15 (68.2)	20 (58.8)	
	2+	2 (9.1)	5 (14.7)	
Condom used at last sexual intercourse		9 (40.9)	15 (44.1)	0.81
Sex during a woman's menses		4 (19.1)	4 (13.8)	0.71
Sex with a woman the same day as meeting		11 (50.0)	9 (26.5)	0.07
Gifts or money in exchange for sex		5 (23.8)	6 (19.4)	0.70
Cleaned penis≤1 hour after last sex		4 (18.2)	10 (30.3)	0.31
Performed oral sex on a female partner		3 (8.3)	1 (4.4)	>0.99
Performed anal sex on a female partner		1 (4.6)	1 (3.0)	0.77
Penis ever feels sore after sex		14 (66.7)	15 (44.1)	0.10
Penis ever gets scratches or cuts during sex		15 (71.4)	19 (55.9)	0.25
Skin of penis ever bleeds during or after sex		5 (23.8)	7 (20.6)	0.78
Any injury to penis during or after sex		18 (85.7)	23 (67.7)	0.21
Ulcer location∧	Prepuce	11 (47.8)	17 (47.2)	0.96
	Glans	3 (13.0)	6 (16.7)	>0.99
	Coronal sulcus	7 (30.4)	12 (33.3)	0.82
	Proximal shaft	1 (4.4)	1 (2.8)	>0.99
	Distal shaft	1 (4.4)	2 (5.6)	>0.99
	Scrotum	1 (4.4)	0 (0.0)	0.39

Not all cells sum to N due to missing data.

+STI-related Etiology indicates ulcers that were associated with HSV or *T. pallidum* by serology or PCR.

* Comparisons by group by Chi-square test, or by Fisher's exact test when cell size <5.

Recall period for behavioral variables are past 6 months unless stated otherwise and are visit-matched to the visit at which GUD was detected.

∧ Ulcer location not mutually exclusive.

### Bacterial Community Comparison by Ulcer Etiology

Overall, 83 distinct genera were detected through massively parallel multitag pyrosequencing analysis of bacteria SSU rRNA genes. Sequences corresponding to *Prevotella* spp. were most abundant, accounting for 18% of microbiota on average, and present in 75% of specimens. The next most abundant bacterial sequences were *Anaerosphaera* spp., *Anaerococcus* spp., *Porpyhromonas* spp., *Fusobacterium* spp., and *Sneathia* spp., accounting for 5–7% of abundance on average, and present in 64.4%, 62.7%, 49.2%, 44.1%, and 44.1% of specimens, respectively (Table 2). Though in low abundance, *Peptoniphilus* spp. was present in 71.2% of specimens and *Dialister* spp. in 44.1%.

**Table 2:** Presence and Average Relative Abundance by Ulcer Etiology with Results of Bray-Curtis Dissimilarity Comparison.

Bacteria (Genus)	Presence by GUD Etiology		Average Relative Abundance by GUD Etiology		Average Dissimilarity	Grouped by Hierarchical Clustering+
	STI-Related, N = 36	Unknown, N = 23	STI-Related	Unknown		
	n (%)	n (%)	%	%		
*Prevotella*	27 (75.0)	17 (73.9)	0.17	0.14	9.80	1
*Fusobacterium*	13 (36.1)	13 (56.5)	0.04	0.07	5.17	2
*Sneathia*	14 (38.9)	12 (52.2)	0.05	0.07	5.12	2
*Anaerococcus*	18 (50.0)	19 (82.6)	0.05	0.06	5.11	
*Anaerosphaera*	21 (58.3)	17 (73.9)	0.05	0.07	4.41	3
*Porphyromonas*	15 (41.7)	14 (60.9)	0.04	0.05	3.89	3
*Escherichia/Shigella*	10 (27.8)	5 (21.7)	0.05	0.01	3.54	4
*Corynebacterium*	10 (27.8)	5 (21.7)	0.04	0.01	3.12	
*Anaerotruncus*	3 (8.3)	3 (13.0)	0.02	0.03	2.92	5*
*Peptostreptococcus*	16 (44.4)	10 (43.5)	0.03	0.03	2.90	
*Staphylococcus*	3 (8.3)	4 (14.4)	0.03	0.02	2.48	4
*Peptoniphilus*	25 (69.4)	17 (73.9)	0.03	0.04	2.40	
*Finegoldia*	10 (27.8)	8 (34.8)	0.02	0.01	1.85	
*Cyanobacter Family 1 Group 1*	11 (30.6)	4 (17.4)	0.02	0.01	1.84	
*Enterobacter*	8 (22.2)	1 (4.4)	0.03	0.00	1.75	4
*Parvimonas*	9 (25.0)	11 (47.8)	0.01	0.02	1.50	
*Propionibacterium*	6 (16.7)	6 (26.1)	0.00	0.02	1.49	
*Campylobacter*	11 (30.6)	7 (30.4)	0.01	0.01	1.25	
*Streptococcus*	5 (13.9)	4 (17.4)	0.01	0.01	1.22	
*Mycoplasma*	5 (13.9)	2 (8.7)	0.01	0.01	1.10	6̂
*Anaerovorax*	7 (19.4)	8 (34.8)	0.01	0.01	1.10	7
*Catonella*	3 (8.3)	3 (13.0)	0.00	0.01	1.03	
*Dialister*	17 (47.2)	9 (39.1)	0.01	0.01	0.87	1
*Oxobacter*	5 (13.9)	7 (30.4)	0.01	0.01	0.85	7
*Soehngenia*	10 (27.8)	9 (39.1)	0.01	0.01	0.65	3
*Sphingomonas*	4 (11.1)	1 (4.4)	0.01	0.00	0.64	4
*Citrobacter*	5 (13.9)	1 (4.4)	0.01	0.00	0.63	4

**Figure 1:**
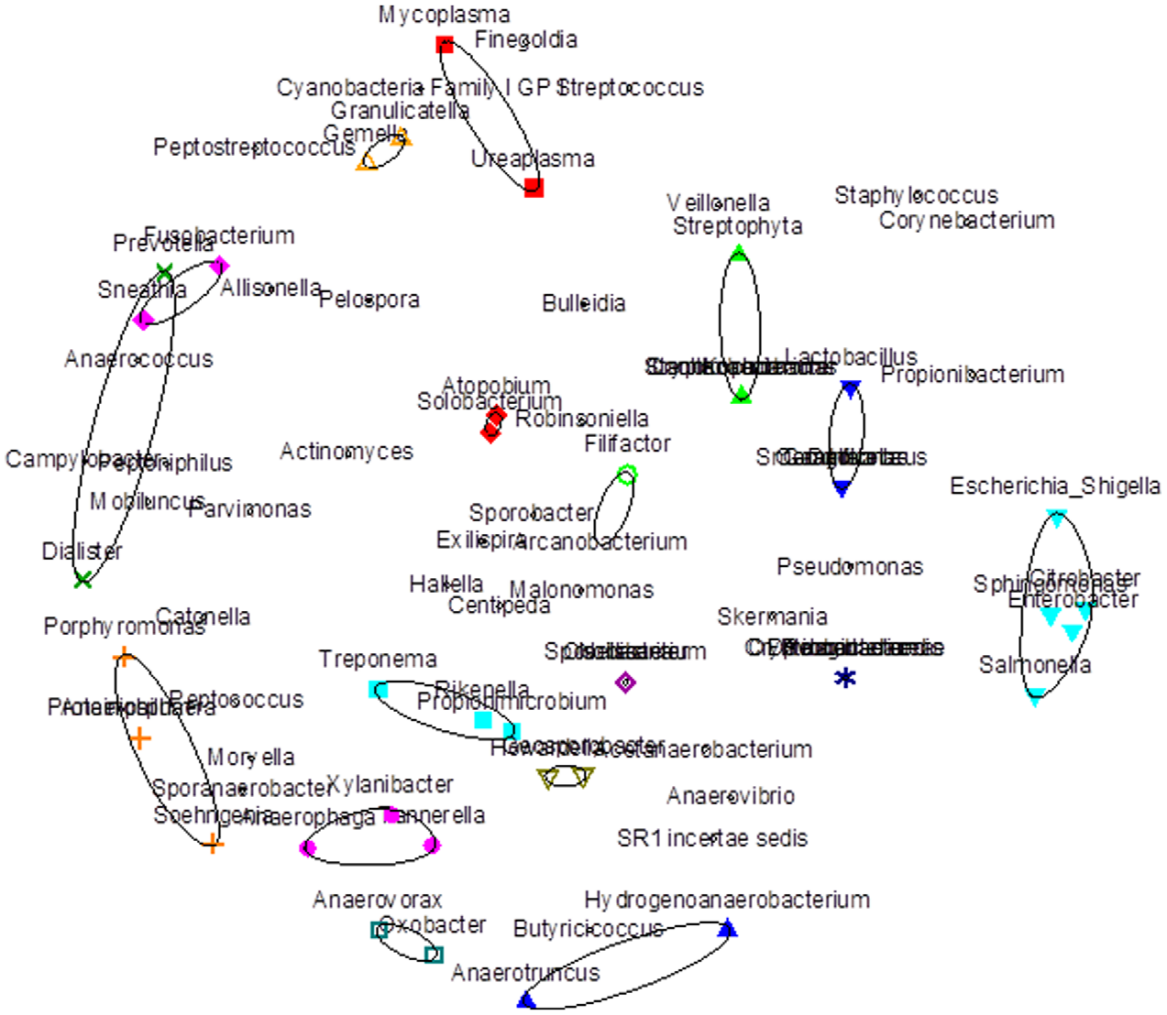
Non-metric multidimensional scaling plot ordinating the correlation in relative abundance between the 83 bacteria identified by pyrosequencing of genital ulcer specimens. To determine if the presence of different Genera of bacteria were associated, hierarchical clustering analysis was performed. The correlation between bacteria is measured by the Spearman rank correlation coefficient; a cutoff of >0.5 was applied as the criterion to differentiate the clusters. Clusters are designated as indicated by green lines encircling bacteria.

* Other group member not identified in bacterial community comparison was Hydrogenoanaerobacterium. ^Other group member not identified in bacterial community comparison was Ureaplasma.

+ Groups identified by hierarchical clustering (Figure 1). Bacteria without group numbers did not cluster with any other bacteria, as defined as a Spearman rank correlation coefficient >0.5.

Bacterial diversity was greater in GUD of unknown etiology than STI-associated GUD (median number of genera 13 [range 7–20] vs. 11 [range 3–20], p = 0.06). The average Shannon-Wiener index, calculated from clustered SSU rRNA gene amplicon sequences (97%), was 3.11 for STI-associated GUD and 2.67 for GUD of unknown etiology (p = 0.01), indicating lower species richness for STI-associated GUD. Comparison of the Bray-Curtis dissimilarity indices by ulcer etiology showed relative homogeneity in the microbiota (step 1 of analysis; Table 2). On average, the Bray-Curtis dissimilarity index was 76.5 (i.e., 23.5% similarity) in genital ulcer associated microbiota. Average similarity was 20.4% among samples from ulcers with STI-associated etiology and 27.8% among samples from ulcers with unknown etiology. Bacteria contributing most to the Bray-Curtis dissimilarity index (Table 1) were *Prevotella* spp. (9.80), *Fusobacterium* spp. (5.17), *Sneathia* spp. (5.12), *Anaerococcus* spp. (5.11), and *Anaerosphaera* spp. (4.41). The relative abundance and/or presence of three individual bacteria were associated with ulcer etiology at the p≤0.10 level (Table 3). *Anaerococcus* spp. and *Parvimonas* spp. were more often detected in ulcers of unknown etiology, and *Enterobacter* spp. was detected more frequently from STI-related ulcers.

**Table 3:** Individual Bacteria and Groups of Bacteria examined by Genital Ulcer Disease etiology.

	Presence by Genital Ulcer Disease Etiology		
Bacteria	STI-related Etiology	Unknown Etiology	Unadjusted OR [95% CI], p-value
	N = 36	N = 23	
	n (%)	n (%)	
*Anaerococcus* spp.	18 (50.0)	19 (82.6)	4.75 [1.33–16.9], 0.02
*Enterobacter* spp.	8 (22.2)	1 (4.4)	0.16 [0.02–1.39], 0.10
*Parvimonas* spp.	9 (25.0)	11 (47.8)	2.75 [0.90–8.45], 0.08
Group 1: *Oxobacter* or *Anaerovorax*	8 (22.2)	10 (43.5)	2.69 [0.85–8.49], 0.09
Group 1: *Oxobacter* and *Anaerovorax*	4 (11.1)	5 (21.7)	2.22 [0.52–9.46], 0.28
Group 1: *Oxobacter* spp. only	7 (30.4)	5 (13.9)	2.71 [0.73–10.0], 0.14
Group 1: *Anaerovorax* spp. only	7 (19.4)	8 (34.8)	2.21 [0.66–7.34], 0.20
Group 2: *Fusobacterium* or *Sneathia*	14 (38.9)	17 (73.9)	4.45 [1.40–14.2], 0.01
Group 2: *Fusobacterium* and *Sneathia*	13 (36.1)	8 (34.8)	0.94 [0.31–2.85], 0.92
Group 2: *Fusobacterium* spp. only	13 (36.1)	13 (56.5	2.30 [0.78–6.76], 0.1
Group 2: *Sneathia* spp. *only*	14 (38.0)	12 (52.2)	1.71 [0.59–4.98], 0.32
Neither Group1 nor Group 2	18 (50.0)	1 (4.4)	Reference
Group 1 only	4 (11.1)	5 (21.7)	22.5 [1.99–254], 0.01
Group 2 only	10 (27.8)	12 (52.2)	21.6 [2.39–195], <0.01
Both Group 1 and Group 2	4 (11.1)	5 (21.7)	22.5 [1.99–254], 0.01

OR  =  Odds Ratio; CI  =  Confidence Interval.

Wald p-values are presented.

### Bacterial Groups Identified by Hierarchical Clustering

Hierarchical clustering (step 2) identified 17 groupings of bacteria with Spearman rank correlation >0.50 (Figure 1). Bacterial members of 4 groups were fully represented and 2 groups were partially represented in the bacterial community analysis in step 1 (Table 2). The relative abundance and presence of these groups of bacteria that were also represented in step 1 were compared by ulcer etiology (Table 3). Two groups of bacteria were associated with ulcer etiology: the grouping of *Oxobacter* spp. and *Anaerovorax* spp., and the grouping of *Fusobacterium* spp. and *Sneathia* spp., the only two members of the Fusobacteria class detected. The presence and relative abundance of *Fusobacterium* spp. and *Sneathia* spp. were each elevated among men with unknown GUD etiology (Table 3), but this did not reach statistical significance. When considered as presence of either bacteria (73.9% unknown etiology vs. 38.9% STI etiology, p = 0.009) or total relative abundance of both bacteria (8.8% vs. 0%, p = 0.024), the differences in Fusobacteria by ulcer etiology were statistically significant. No dose response pattern was detected using 1% and 10% cutoffs for either bacteria group, and no threshold for increased risk was identified through sensitivity analysis [results not shown].

### Multivariable Logistic Regression: Factors Associated with Ulcers of Unknown Etiology

In multivariable logistic regression analysis, only age and presence of any Fusobacteria remained statistically significant at the p<0.05 level (Table 4). Men with ulcers of unknown etiology were more likely to be aged 21–24 [adjusted OR (aOR)  = 3.90; 95% CI: 1.10–13.9] and more likely to have recovery of Fusobacteria [aOR  = 5.67; 95% CI: 1.63–19.8]. The increased odds of recovery of *Oxobacter* spp. or *Anaerovorax* spp. remained marginally statistically significant [aOR = 3.12; 95% CI: 0.83–11.7]. While *Anaerococcus* spp. were no longer significant when adjusted for Fusobacteria, *Parvimonas* spp. and *Enterobacter* spp. also were not significant when adjusted for age or Fusobacteria. Reporting that the “penis ever feels sore during sex” remained marginally significant when controlling for Fusobacteria, but was no longer significant when controlling for age. Exclusion of the 14 men who were HSV-2 seropositive but did not have HSV or *T. pallidum* recovered from the ulcer by PCR resulted in the same final multivariable model with similar magnitude of odds ratios for age [aOR = 3.82; 95% CI: 0.91–16.0] and Fusobacteria [aOR = 6.10; 95% CI: 1.32–28.2], though the association between recovery of *Oxobacter* spp. or *Anaerovorax* spp. was strengthened [aOR = 6.40; 95% CI: 1.10–37.5].

**Table 4:** Results of Multivariable Logistic Regression: Factors Associated with Genital Ulcers of Unknown Etiology.

Variable		Unadjusted OR [95% CI], p-value	Adjusted OR [95% CI], p-value
Age in years at Baseline	18–20	Reference	Reference
	21–24	3.75 [1.23–11.4], 0.020	3.90 [1.10–13.9], 0.035
Bacteria groups	Oxobacter and/or Anaerovorax	2.69 [0.85–8.49], 0.091	3.12 [0.83–11.7], 0.092
	Fusobacterium and/or Sneathia	4.45 [1.40–14.2], 0.011	5.67 [1.63–19.8], 0.006
Reported penis ever sore during or after sex in the last 6 months		2.53 [0.81–7.94], 0.011	
Reported having sex with a woman the same day as meeting her		2.78 [0.89–8.70], 0.079	

Ref  =  reference group; OR  =  Odds Ratio; CI  =  Confidence Interval.

Adjusted ORs and 95% CIs presented for variables significant Wald p-value <0.10.

### Factors Associated with Bacteria Associated with Ulcers of Unknown Etiology

To further elucidate the potential mechanism by which these bacteria were more commonly detected in GUD with unknown etiology, we compared socio-demographic and behavioral characteristics by bacterial recovery (Table 5). Detection of sequences derived from *Oxobacter* spp. and/or *Anaerovorax* spp. was more common among uncircumcised than circumcised men (100% vs. 78%, p = 0.046), as was Fusobacteria (93.6% vs. 75.0%, p = 0.071). Detection of these bacteria was not associated with any sexual behaviors, though reported bleeding of the skin of the penis was more common among men with *Oxobacter* spp. and/or *Anaerovorax* spp. (33.3% vs. 13.5%, p = 0.085).

**Table 5:** Factors Associated with Select Bacterial Groups.

		*Oxobacter* and/or *Anaerovorax* recovered from genital ulcer specimen		*Fusobacterium* and/or *Sneathia* recovered from genital ulcer specimen	
		No, N = 41	Yes, N = 18	No, N = 28	Yes, N = 31
		n (%)	N (%)	n (%)	n (%)
Age in years at baseline	18–20	24 (58.5)	8 (44.4)	16 (57.1)	16 (51.6)
	21–24	17 (41.5)	10 (55.6)	12 (42.9)	15 (48.4)
Married/cohabiting		3 (8.1)	2 (11.1)	2 (7.7)	3 (10.3)
Uncircumcised		32 (78.0)	18 (100.0)*	21 (75.0)	29 (93.6)^
HIV positive		3 (8.1)	1 (5.6)	0 (0.0)	4 (14.3)
Penis ever gets scratches or cuts during sex		22 (59.5)	12 (66.7)	17 (63.0)	17 (60.7)
Skin of penis ever bleeds during or after sex		5 (13.5)	7 (38.9)*	9 (33.3)	3 (10.7)^
Penis ever feels sore after sex		18 (48.7)	11 (61.1)	14 (51.9)	15 (53.6)
2 or more sex partners past 30 days		4 (10.5)	3 (16.7)	2 (7.4)	5 (17.2)
Sex with a woman during her menses		4 (12.5)	4 (22.2)	4 (18.2)	4 (14.3)
Sex with a woman the same day as meeting her		12 (31.6)	9 (50.0)	8 (29.6)	12 (41.4)
Sex with a woman in exchange for gifts or money		7 (20.6)	4 (22.2)	4 (16.7)	7 (25.0)
Used a condom at last sexual intercourse		17 (44.7)	7 (38.9)	13 (48.2)	11 (37.9)
Cleaned penis≤ 1 hour after last sexual intercourse		8 (21.6)	6 (33.3)	6 (23.1)	8 (27.6)

Not all cells sum to N due to missing data.

Comparisons by group by Chi-square test, or by Fisher's exact test when cell size <5.

Statistical significance indicated by (*) or (∧):

* p-value <0.05.

∧ 0.05< p-value <0.10.

## Discussion

Among 59 clinician-detected genital ulcers, 23 (39%) did not have ulcerative STI pathogen recovered from the ulcer or diagnosed by serology. This proportion of genital ulcers without STI etiology is high, but in keeping with findings from other studies. Observational studies with STI testing have observed the proportion of genital ulcers with unknown etiology in men and women to be 27–39% in Kenya [17], South Africa [18]–[19], Botswana [20], Madagascar [21], and Tanzania [22].

We detected four bacterial taxa (*Fusobacterium, Sneathia, Oxobacter* and *Anaerovorax*) occurring in two groups that were more likely to be detected in genital ulcers of unknown etiology. *Fusobacterium* spp. and *Sneathia* spp. are anaerobic bacteria that are strongly associated with Bacterial vaginosis (BV) [23]–[24]. We found no published studies relating them to genital ulcers. However, a number of strains of *Fusobacterium* spp. have been associated with periodontal disease and oral mucosal ulcers [25]–[27]. The mechanism by which this is thought to occur is through induction of cytokines and other effector molecules with inflammatory and tissue-destroying capabilities [27]. It is possible that men in our study acquired these bacteria from female sex partners through vaginal or oral sex, or from their own genital tract. Coupled with pre-existing epithelial disruptions, this may have led to an ulcer-like appearance in some men. *Oxobacter* spp. and *Anaerovorax* spp. are strictly anaerobic members of the Clostridiales family. Only one species of the genus *Oxobacter*, *Oxobacter pfennigi* (previously *Clostridium pfennigii*), has been validly described [28], and we found no published data in animals or humans relaying its biological function. The only described species of the genus *Anaerovorax* is *A. odorimutans*, which derives its name from its fermenting of putrescine, an amine associated with malodor in cadavers and BV [29]. With a paucity of data and lack of cultivation of these bacteria, their biological function is largely unknown. Results such as ours, relating the microbiota to clinical and behavioral data, may help elucidate the biological activities of these bacteria. Studies are needed to examine inter-relatedness or co-dependence of these organisms. The difficulty in developing hypotheses regarding disease etiology by correlating limited taxa information with clinical presentation highlights the need for multifaceted studies that include directed isolation attempts to recover key taxa of interest, coupled with physiological testing and genome sequencing, genome reconstruction, and meta-transcriptome analyses to identify *in situ* activity of key taxa prior to and after development of GUD.

Circumcision status was not associated with GUD of unknown etiology, but uncircumcised men were more likely to have bacteria that were associated with GUD of unknown etiology. Prior studies have demonstrated that uncircumcised men are more likely to harbor anaerobic bacteria in the penile microbiota [16], [30]. From our previous analysis, uncircumcised men are more likely to have epithelial disruptions of the penile skin, likely to be mechanical in origin [31]. It is possible that anaerobic bacteria are simply more likely to colonize pre-existing epithelial disruptions. However, many of the anaerobic bacteria detected in the uncircumcised men in this study, such as *Prevotella* spp., *Dialister* spp., *and Anaerococcus* spp., were not associated with GUD of unknown etiology, supporting the specificity of our findings. The cross-sectional nature of the study does not allow us to distinguish between causal effects and colonization, but this is unlikely to be a simple “either-or” situation: we postulate that these bacteria may be colonizing *and* exacerbating minor epithelial disruptions, leading to an ulcer-like appearance.

The four bacteria associated with GUD of unknown etiology were not completely specific to men with GUD of unknown etiology, indicating they are unlikely to be uniformly pathogenic. Genital ulceration associated with these bacteria may require an unmeasured co-factor. We did not find sexual behaviors that were associated with recovery of these bacteria, though power to detect modest differences was limited. Men who were aged 21–24 at baseline were more likely to have GUD of unknown etiology than those aged 18–20. This difference may reflect a longer duration of being sexually active, different selection of sexual partners, different genital hygiene practices, or unconsidered environmental factors. We did not have a measure of time since last sexual intercourse, and the association between sexual events and practices occurring more remotely may have been attenuated. Future study should assess the event-level timing of specific sexual and hygiene practices in relation to genital ulcers to attempt to assess the temporal association between behaviors, bacteria, and symptoms.

The use of multitag pyrosequencing enabled a comprehensive assessment of potentially associated bacterial pathogens, and did not identify any additional cases of *T. pallidum* or *H. ducreyi* within our cohort, or other ulcerative STI pathogens (*C. trachomatis* lymphogranuloma venereum or *C. granulomatis*). We did not examine non-bacterial pathogens. Female genital schistosomiasis affects 9–13 million women worldwide [32], with 35–75% of infected women manifesting genital ulcers or erosions of the vulva, vagina, or cervix [33]–[34]. We did not find published reports of schistosomiasis-associated GUD in men. This is a potential cause to be explored, considering the location of Kisumu on the shores of Lake Victoria and the endemicity of schistosomiasis in the surrounding area [35]. We are unaware of non-herpetic viruses as a potential likely cause of genital ulcers in men, though there are case reports of Epstein Barr Virus (EBV) recovery from genital ulcers in women [36]. In a study of 45 uncircumcised men, PCR detected EBV in 13% of coronal sulcus specimens, but this was not associated with ulcers and there was no comparison to circumcised men [37]. It is possible GUD of unknown etiology may have been non-infectious. Genital dermatoses such as psoriasis, erosive lichen planus, and plasma cell balanitis are more common among uncircumcised men [38], and may have been misclassified as genital ulcers in our study. This higher preponderance of non-herpetic and non-syphilitic “ulcers” in uncircumcised men would explain our finding of circumcision's protective effect on GUD, with no effect on HSV-2 or syphilis incidence.

### Limitations

Prospective study is necessary to distinguish the extent to which the bacteria which were associated with genital ulcers of unknown etiology are causal and/or colonizing. More definitive knowledge of the depth and function of bacterial invasion of these ulcers would require curettage. Given the genital location of the ulcers, this likely presents unjustified risk in obtaining tissue specimens. Study of men seeking circumcision may be able to examine bacterial invasion in removed foreskin, although men are generally deferred from medical circumcision if they have active ulcers or herpetic lesions on the penis. While pyrosequencing indicates the types of bacteria present, we don't have a measure of absolute numbers of bacteria in the ulcers, and bacterial load may be an important explanatory factor in which men develop ulcers and which do not. Replication of our findings is an essential next step.

Though this pilot study had a limited sample size, to our knowledge it represents the largest study in the published literature of the penile microbiota utilizing multitag pyrosequencing. This is the first study to broadly examine genital ulcers for non-STI bacterial pathogens. We defined GUD to be of HSV-2 related etiology if the subject was positive for HSV-2 by PCR or serology, due to variability in the point in time of the natural history of the ulcer at which the specimen was obtained. However, it is possible that the 12 subjects who were HSV-2 seropositive but whose ulcers were HSV negative by PCR could have had ulcers of different etiology. For this reason, we conducted analyses including and excluding men, and results were similar. Although we were unable to test all men with clinically observed GUD, those selected for PCR and pyrosequencing did not differ from those who were not tested with regards to behavioral risks or STI history, indicating that resource constraints did not produce significant selection bias. However, in our sample incident GUD was underrepresented and the etiology and factors associated with incident GUD, especially circumcision status, may have differed.

## Conclusions

Our results provide a new view of genital ulcer disease. Understanding the etiology of GUD is necessary for effective treatment and prevention, to understand the mechanism by which it is reduced by circumcision, and for reduction as a cofactor for HIV acquisition and transmission. Prospective investigation of genital microbial profiles, behaviors leading to specific genital milieu, inflammation, and GUD are needed to verify the nature and causes of clinically detected genital ulcers. Analysis of these relationships with high quality data is lacking in the published literature. Such study may lead to more effective approaches to preventing and treating GUD and could provide insight into potential mechanisms for increased risk of HIV acquisition.

## References

[1] Auvert B, Taljaard D, Lagarde E, SobNGwi-Tambekou J, Sitta R, et al (2005) Randomized, controlled intervention trial of male circumcision for reduction of HIV infection risk: the ANRS 1265 Trial. PLoS Med 2(11): e298. 16231970 10.1371/journal.pmed.0020298 PMC1262556

[2] Gray RH, Kigozi G, Serwadda D, Makumbi F, Watya S, et al (2007) Male circumcision for HIV prevention in men in Rakai, Uganda: a randomised trial. Lancet 369: 657–66. 17321311 10.1016/S0140-6736(07)60313-4

[3] Bailey RC, Moses S, Parker CB, Agot K, Maclean I, et al (2007) Male circumcision for HIV prevention in young men in Kisumu, Kenya: a randomised controlled trial. Lancet 369: 643–56. 17321310 10.1016/S0140-6736(07)60312-2

[4] Tobian AA, Serwadda D, Quinn TC, Kong X, Oliver A, et al (2009) Male circumcision for the prevention of HSV-2 and HPV infections and syphilis. N Engl J Med 360: 1298–1309. 19321868 10.1056/NEJMoa0802556 PMC2676895

[5] Sobngwi-Tambekou J, Taljaard D, Lissouba P, Zarca K, Puren A, et al (2009) Effect of HSV-2 serostatus on acquisition of HIV by young men: results of a longitudinal study in Orange Farm, South Africa. J Infect Dis199: 958–64. 10.1086/597208 PMC2868899 19220143

[6] Gray RH, Serwadda D, Tobian AA, Chen MZ, Makumbi F, et al (2009) Effects of genital ulcer disease and herpes simplex virus type 2 on the efficacy of male circumcision for HIV prevention: Analyses from the Rakai trials. PLoS Med 6(11): e1000187. 19936044 10.1371/journal.pmed.1000187 PMC2771764

[7] Mehta SD, Moses S, Parker CB, Agot K, Maclean I, et al. (2012) The protective effect of medical male circumcision against HIV is not mediated by reduction in HSV-2: Results from the randomized trial of MMC to reduce HIV in Kisumu, Kenya. *AIDS.*. In press.

[8] Wald A, Link K (2002) Risk of human immunodeficiency virus infection in herpes simplex virus type 2-seropositive persons: a meta-analysis. J Infect Dis 185: 45–52. 11756980 10.1086/338231

[9] Freeman EE, Weiss HA, Glynn JR, Cross PL, Whitworth JA, et al (2006) Herpes simplex virus 2 infection increases HIV acquisition in men and women: systematic review and meta-analysis of longitudinal studies. AIDS 20: 73–83. 16327322 10.1097/01.aids.0000198081.09337.a7

[10] Celum CL (2004) The interaction between herpes simplex virus and human immunodeficiency virus. Herpes 11 Suppl 136A–45A. 15115628

[11] Celum C, Wald A, Lingappa JR, Magaret AS, Wang RS, et al (2010) Acyclovir and transmission of HIV-1 from persons infected with HIV-1 and HSV-2. N Engl J Med 362: 427–39. 20089951 10.1056/NEJMoa0904849 PMC2838503

[12] Paz-Bailey G, Sternberg M, Puren AJ, Steele L, Lewis DA (2010) Determinants of HIV type 1 shedding from genital ulcers among men in South Africa. Clin Infect Dis50: 1060–7. 10.1086/651115 20178417

[13] Spear GT, Gilbert D, Sikaroodi M, Doyle L, Green L, et al (2010) Identification of rhesus macaque genital microbiota by 16S pyrosequencing shows similarities to human bacterial vaginosis: implications for use as an animal model for HIV vaginal infection. AIDS Res Hum Retroviruses 26: 193–200. 20156101 10.1089/aid.2009.0166 PMC2835387

[14] Cole JR, Chai B, Farris RJ, Wang Q, Kulam SA, et al (2005) The Ribosomal Database Project (RDP-II): Sequences and tools for high-throughput rRNA analysis. Nucleic Acids Res 33: 294–296. 10.1093/nar/gki038 PMC539992 15608200

[15] Gihring TM, Green SJ, Schadt CS (2012) Massively parallel rRNA gene sequencing exacerbates the potential for biased community diversity comparisons due to variable library sizes. Environ Microbiol 14: 285–290. 21923700 10.1111/j.1462-2920.2011.02550.x

[16] Price LB, Liu CM, Johnson KE, Aziz M, Lau MK, et al (2010) The effects of circumcision on the penis microbiota. PLoS One 5: e8422. 20066050 10.1371/journal.pone.0008422 PMC2798966

[17] Plummer FA, D'Costa LJ, Nsanze H, Karasira P, MacLean IW, et al (1985) Clinical and microbiologic studies of genital ulcers in Kenyan women. Sex Transm Dis 12: 193–7. 3878601 10.1097/00007435-198510000-00005

[18] Moodley P, Sturm PD, Vanmali T, Wilkinson D, Connolly C, et al (2003) Association between HIV-1 infection, the etiology of genital ulcer disease, and response to syndromic management. Sex Transm Dis 30: 241–5. 12616144 10.1097/00007435-200303000-00013

[19] O'Farrell N, Hoosen AA, Coetzee KD, van den Ende J (1992) Sexual behaviour in Zulu men and women with genital ulcer disease. Genitourin Med 68: 245–8. 1398660 10.1136/sti.68.4.245 PMC1194882

[20] Paz-Bailey G, Rahman M, Chen C, Ballard R, Moffat HJ, et al (2005) Changes in the etiology of sexually transmitted diseases in Botswana between 1993 and 2002: implications for the clinical management of genital ulcer disease. Clin Infect Dis 41: 1304–12. 16206106 10.1086/496979

[21] Behets FM, Andriamiadana J, Randrianasolo D, Randriamanga R, Rasamilalao D, et al (1999) Chancroid, primary syphilis, genital herpes, and lymphogranuloma venereum in Antananarivo, Madagascar. J Infect Dis 180: 1382–5. 10479178 10.1086/315005

[22] Ahmed HJ, Mbwana J, Gunnarsson E, Ahlman K, Guerino C, et al (2003) Etiology of genital ulcer disease and association with human immunodeficiency virus infection in two Tanzanian cities. Sex Transm Dis 30: 114–9. 12567167 10.1097/00007435-200302000-00004

[23] Ling Z, Kong J, Liu F, Zhu H, Chen X, et al (2010) Molecular analysis of the diversity of vaginal microbiota associated with bacterial vaginosis. BMC Genomics 11: 488. 20819230 10.1186/1471-2164-11-488 PMC2996984

[24] Spear GT, Sikaroodi M, Zariffard MR, Landay AL, French AL, et al (2008) Comparison of the diversity of the vaginal microbiota in HIV-infected and HIV-uninfected women with or without bacterial vaginosis. J Infect Dis 198: 1131–40. 18717638 10.1086/591942 PMC2800037

[25] Brook I (2004) Microbiology and management of peritonsillar, retropharyngeal, and parapharyngeal abscesses. Oral Maxillofac Surg 62: 1545–50. 10.1016/j.joms.2003.12.043 15573356

[26] Enwonwu CO, Falkler WA Jr, Idigbe EO, labi BM, Ibrahim M, et al (1999) Pathogenesis of cancrum oris (noma): confounding interactions of malnutrition with infection. Am J Trop Med Hyg 60: 223–32. 10072140 10.4269/ajtmh.1999.60.223

[27] Wilson M (1995) Biological activities of lipopolysaccharides from oral bacteria and their relevance to the pathogenesis of chronic periodontitis. Sci Prog 78: 19–34. 7597416

[28] Collins MD, Lawson PA, Willems A, Cordoba JJ, Fernandez-Garayzabal J, et al (1994) The phylogeny of the genus Clostridium: proposal of five new genera and eleven new species combinations. Int J Syst Bacteriol 44: 812–26. 7981107 10.1099/00207713-44-4-812

[29] Wolrath H, Forsum U, Larsson PG, Borén H (2001) Analysis of bacterial vaginosis-related amines in vaginal fluid by gas chromatography and mass spectrometry. J Clin Microbiol 39: 4026–31. 11682525 10.1128/JCM.39.11.4026-4031.2001 PMC88482

[30] Nelson DE, Van Der Pol B, Dong Q, Revanna KV, Fan B, et al (2011) Charcteristic male urine microbiotas associate with asymptomatic sexually transmitted infection. PLoS One 5(11): e14116. 10.1371/journal.pone.0014116 PMC2991352 21124791

[31] Mehta SD, Krieger JN, Agot K, Moses S, Ndinya-Achola JO, et al (2010) Circumcision and reduced risk of self-reported coital injuries: Results from a randomized controlled trial in Kisumu, Kenya. J Urol 184: 203–9. 20483156 10.1016/j.juro.2010.03.015 PMC3090633

[32] Feldmeier H, Poggensee G, Krantz I, Helling-Giese G (1995) Femalegenital schistosomiasis. New challenges from a gender perspective. Trop Geographical Med47: 2–15. 7618212

[33] Poggensee G, Kiwelu I, Weger V, Göppner D, Diedrich T, et al (2000) Female genital schistosomiasis of the lower genital tract: prevalence and disease-associated morbidity in northern Tanzania. J Infect Dis 181: 1210–3. 10720558 10.1086/315345

[34] Swai B, Poggensee G, Mtweve S, Krantz I (2006) Female genital schistosomiasis as an evidence of a neglected cause for reproductive ill-health: a retrospective histopathological study from Tanzania. BMC Infect Dis 6: 134. 16928276 10.1186/1471-2334-6-134 PMC1564144

[35] Opisa S, Odiere MR, Jura WG, Karanja DM, Mwinzi PN (2011) Malacological survey and geographical distribution of vector snails for schistosomiasis within informal settlements of Kisumu City, western Kenya. Parasit Vectors 4: 226. 22152486 10.1186/1756-3305-4-226 PMC3247081

[36] Halvorsen JA, Brevig T, Aas T, Skar AG, Slevolden EM, et al (2006) Genital ulcers as initial manifestation of Epstein-Barr virus infection: two new cases and a review of the literature. Acta Derm Venereol 86: 439–42. 16955191 10.2340/00015555-0140

[37] Näher H, Gissmann L, Freese UK, Petzoldt D, Helfrich S (1992) Subclinical Epstein-Barr virus infection of both the male and female genital tract – indication for sexual transmission. J Invest Dermatol 98: 791–3. 1314867 10.1111/1523-1747.ep12499958

[38] Mallon E, Hawkins D, Dinneen M, Francis N, Fearfield L, et al (2000) Circumcision and genital dermatoses. Arch Dermatol 136: 350–354. 10724196 10.1001/archderm.136.3.350

